# Development of a remotely controllable 4 m long aerial-hose-type firefighting robot

**DOI:** 10.3389/frobt.2023.1273676

**Published:** 2023-12-22

**Authors:** Yu Yamauchi, Yukihiro Maezawa, Yuichi Ambe, Masashi Konyo, Kenjiro Tadakuma, Satoshi Tadokoro

**Affiliations:** ^1^ Faculty of Systems Science and Technology, Akita Prefectual University, Yurihonjyo, Japan; ^2^ Graduate School of Information Sciences, Tohoku University, Sendai, Japan; ^3^ Graduate School of Engineering Science, Osaka University, Toyonaka, Japan; ^4^ Tough Cyberphysical AI Research Center, Tohoku University, Sendai, Japan

**Keywords:** firefighting robot, continuum robot, world robot summit, water jet, aerial-hose-type robot, demonstration system

## Abstract

In a fire outbreak, firefighters are expected to rapidly extinguish fires to stop the spread of damage and prevent secondary disasters. We proposed the concept of a dragon firefighter (DFF), which is a flying-hose-type firefighting robot. We developed a 3.6 m long DFF equipped with two nozzle units and achieved stable flight. However, the system was not yet completed because the root of the robot, which should have been operated remotely, was operated manually. In addition, the system’s reliability was insufficient to successfully repeat the demonstration several times. The development of a robot demonstration system is crucial for the practical application of such a firefighting robot. In this study, we developed a demonstration system for a remotely controllable 4 m flying firehose robot for demonstration at the World Robot Summit 2020 (WRS 2020) opening ceremony in Fukushima as a milestone. This paper focuses on the following issues: 1): installation of the remotely controllable mobile base, 2): redesign of the water channels (the sizes of nozzle outlets) to get enough thrusts to fly with a fire engine, 3): development of nozzle units with a larger movable range (1.5 times larger than the conventional nozzle) in addition to waterproofing technique to improve system reliability, and 4): redesign of a passive damping mechanism to ensure better stability. Thus, a firefighting demonstration was successfully conducted at the opening ceremony of the World Robot Summit 2020 in Fukushima, Japan, and we discuss the lessons learned through the demonstration. We found that the developed DFF system incorporating a mobile base could achieve remote fire extinguishing.

## 1 Introduction

In a fire outbreak, firefighters are expected to rapidly extinguish the fire to stop the spread of damage and prevent secondary disasters. Among the firefighting tactics employed, remote water spraying is primarily utilized to suppress the spread of fire in buildings. This approach is the preferred considering the risk for firefighters entering the building, such as potential structural collapse or the generation of toxic gases during a fire. Consequently, the current firefighting tactics require a significant amount of time to fully extinguish a fire.

To address this challenge, many robotic approaches have been developed. For example, Liljebäck et al. developed a prototype of a snake firefighter robot, which is a cable-like, crawling-type robot driven by water pressure, to extinguish fires in hazardous environments, including tunnels (Liljeback et al., 2006). Another notable innovation is the Shark Robotics Colossus firefighting robot, a crawler-type robot equipped with a water spout; it can be remotely controlled to move around, which helps firefighters in extinguishing fires, clearing debris, and gathering information at the fire scene (Peskoe-Yang, 2019). Although Colossus can move over uneven terrain, it can primarily move along the ground and may encounter difficulties in self-propulsion when obstructed by unstable debris, obstacles, or other objects.

Recently, unmanned aerial vehicles (UAVs), such as multi-rotors, have been applied in firefighting tasks. Viegas et al. developed and demonstrated a light-weight tethered UAV with mixed multi-rotor and water jet propulsion for forest fire fighting (Viegas et al., 2022). Some companies also developed and demonstrated tethered UAVs for firefighting for high buildings (Ibekwe, 2018; Wang and Wang, 2020). Lee et al. proposed a stabilized levitation controller for the jet-actuated drone for firefighting (Lee et al., 2021). However, when entering the building, particularly in confined spaces, the connected water hose may interfere with the environment and generate a large drag force.

**FIGURE 1:**
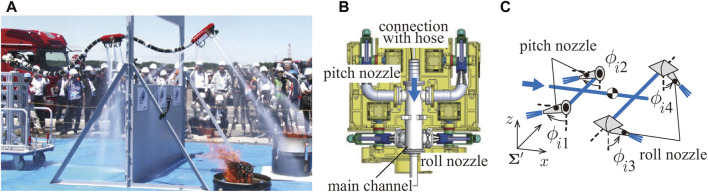
**(A)** Dragon Firefighter. **(B)** Computer-aided design (CAD) of previous nozzle unit. **(C)** Structure of the nozzle unit.

Similarly, we have proposed the concept of an aerial firefighting hose robot known as a Dragon Firefighter (DFF) (Figure 1A). Unlike conventional UAVs, in DFF, the entire water hose can be manipulated using multiple nozzle units distributed on the hose. Our objective is to enable the robot to enter buildings for extinguishing fires safely and rapidly. Particularly, the primary body of the robot comprises a flexible body and nozzle units. Water is supplied into the body and expelled from the nozzle units to achieve flight. Moreover, the water jet can be effectively used to extinguish fires. The robot has a fisheye camera and thermal imaging camera on its tip to identify the location of a fire and apply water directly to the fire source. Therefore, the robot is expected to extinguish fires safely and efficiently using remote control.

Thus far, we have developed a 3.6 m long DFF equipped with two nozzle units and achieved stable flight (Ando et al., 2020). This robot was used in a small-scale firefighting demonstration with a burning surface area of approximately 3.27 *m* ^2^ at the seventh Open Field Evaluation of the ImPACT Tough Robotics Challenge funded by the Council for Science and Technology Policy, Japan. The demonstration involved the DFF going through a 1.5 m high gap simulating a window frame and extinguishing two fires beyond it. Although the demonstration shows the promising potential of the developed robot, the system was not yet completed because the root of the robot, which should have been operated remotely, was moved manually.

Developing a robot demonstration system is crucial for the practical application of such a firefighting robot. Using a demonstration system to publicize the potential of the new technology, it will be easier to discuss its implementation in society. In addition, by conducting several firefighting experiments under realistic conditions using the demonstration system, new firefighting techniques, such as robot specifications and operation methods that will be required in the real world, can be discussed for the next firefighting stage.

In this study, we developed a demonstration system for the remotely controllable 4 m flying firehose robot for demonstration at the World Robot Summit 2020 opening ceremony in Fukushima as a milestone. For successful demonstration, we need some specific developments of the robots. In particular, we focused on the following issues: 1): installation of the remotely controllable mobile base, 2): redesign of water channels (the sizes of nozzle outlets) to get enough thrust to fly with a fire engine, 3): development of nozzle units with a larger range of realized net force in addition to waterproofing technique to improve system reliability, and 4): redesign of a passive damping mechanism to ensure better stability. Finally, a firefighting demonstration was successfully conducted with 4 m flying DFF at the opening ceremony of the World Robot Summit 2020 in Fukushima, Japan, and we discussed the lessons learned through the demonstration.

## 2 Related study

Long hose-type robots can potentially be used to gather information and work by changing their shapes and propelling their bodies. Various driving mechanisms have been proposed for manipulating the flexible bodies of these robots (Walker, 2013). For example, pneumatic-driven mechanisms change the shape of the robot using multiple distributed pneumatic actuators (Greer et al., 2017). Torus-shaped structures for extension and steering have also been proposed (Hawkes et al., 2017). Jet-actuated mechanisms that can directly generate translational forces to steer and propel by expelling fluid jets have recently been developed (Silva Rico et al., 2017; Ishii et al., 2018; Fujikawa et al., 2019; Ambe et al., 2022; Yamauchi et al., 2022a; Yamauchi et al., 2022b; Ambe et al., 2023; Huynh et al., 2023; Maezawa et al., 2023).

In similar studies to our concept, Eberl et al. developed a “water-jet hose manipulation device,” which uses a sleeve-like mechanism that rotates around the flow direction to change the direction of the water jet. It also has a mechanism at the end of the hose to control the jet flow rate. However, they did not realize a stabilized flight with the mechanism (Eberl and Majdic, 2015). To the best of our knowledge, this study is the first to develop a water-jet-actuated continuum robot as firefighting demo systems.

## 3 Conventional DFF

### 3.1 Overview

The following is an overview of DFFs that have been developed so far (Ando et al., 2020). The DFF shown in Figure 1A is approximately 3.6 m long, with nozzle units located at the tip and the middle of the DFF. The end of the DFF is connected to a manual cart, which can be moved back and forth as the cart is pushed.

As shown in Figures 1B, C, each nozzle unit is equipped with four nozzles, which can change the injection direction (two for roll rotations, the others for pitch rotations). Flexible tubes are used to switch the jetting direction with low flow resistance. Therefore, the injection range is limited to the range where the tube does not buckle. The movable range of each injection nozzle is approximately ±60°. In addition, to reduce weight, the actuators mounted in the middle of the nozzle unit responsible for nozzle rotation in the pitch direction has been removed. Notably, the nozzle is designed to be injected directly below its nozzle. The servo motors on the nozzle unit suffer from water leakage due to inadequate waterproofing.

### 3.2 Controller

DFF control is performed for the net force $f_{i}^{n}$ of the nozzle unit *i* as the control input (Ando et al., 2018; Ambe et al., 2023). $f_{i}^{n}$ is the force vector in the inertial coordinate. The controller is specifically represented using Equation 1.

$$f_{i}^{n}=F_{i}^{n}-D_{d_{i}}{\dot{r}}_{i}$$ (1)

$F_{i}^{n}$ is a constant force vector in the inertial coordinate to determine the flying shape (equilibrium point). An operator commands this to change the flying shape. A derivative controller $-D_{d_{i}}{\dot{r}}_{i}$ is also installed in conjunction with the velocity vector of the nozzle unit ${\dot{r}}_{i}$ to dampen vibration. $D_{d_{i}}$ is a 3×3 constant matrix defined by *D*_*d*_ = diag[*s*, *s*, *s*]. In addition, because the nozzle unit has four active nozzles (four degrees of freedom (DOF)), the torsional torque $\tau_{i}^{\text{t}}$ of nozzle unit *i* is determined using a proportional derivative controller for the twist angle (Ando et al., 2020).

**FIGURE 2:**
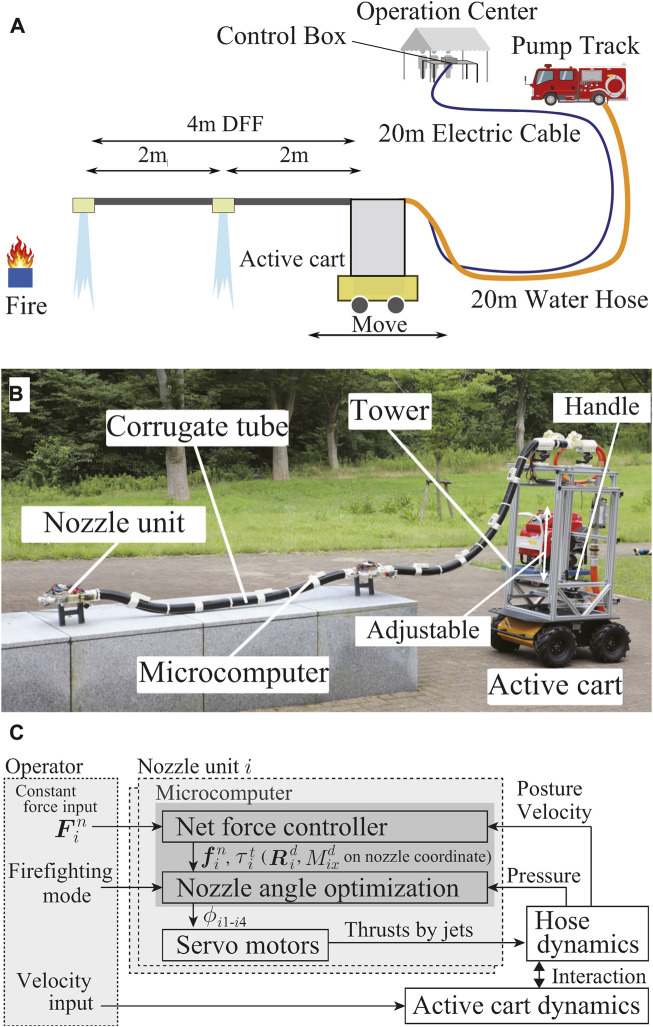
**(A)** Overview of the demonstration at WRS. **(B)** Developmed DFF System. **(C)** Brief control diagram.

To achieve the net force $f_{i}^{n}$ and the torsional torque $\tau_{i}^{\text{t}}$ of the nozzle unit, the directions of the rotating nozzles are determined based on a quadratic programming method (nozzle angle optimization). The details are provided in Section 5.5 and (Ando et al., 2020). Notably, the determined nozzle angles are achieved through position control of the servo motors connected to the rotating nozzles (Figure 2C).

## 4 Overview of the demonstration at the World Robot Summit

### 4.1 World Robot Summit

The World Robot Summit (WRS) includes the World Robot Challenge (WRC), in which teams from around the world compete in various fields regarding robot applications, and the World Robot Expo (WRE), which presents the current and future states of robot utilization. This event provides an ideal scenario where people can connect and discuss the future of robotics. From October 17 to 21, 2018, WRS2018 was held at the Tokyo Big Sight East Hall, as a pre-conference (Tadokoro et al., 2019). WRS2020 Aichi was held at the Aichi Sky Expo (Aichi International Exhibition Center) from September 9 to 12, 2021, and WRS2020 Fukushima was held at the Fukushima Robot Test Field from October 8 to 10, 2021. At the opening ceremony of WRS2020 Fukushima, a demonstration of the torch relay and lighting of the torch stand by multiple robots was performed.

### 4.2 Opening ceremony

The demonstration at the opening ceremony comprised three steps: 1) igniting fireballs by a snake robot, 2) lighting the torch by a robotic arm grasping the fireball, and 3) extinguishing the ignited fireballs by the DFF. [Demonstration video is available on YouTube (World Robot Summit, 2021)]. 1) A snake-like robot developed by Takemori et al. climbed up a ladder and pushed out a ball at the top of the ladder (Takemori et al., 2018). The pushed ball rolled down the slope and ignited (fireball). 2) The robot arm with a fire-resistant hand was operated remotely using the “Successor” robot system developed by Kawasaki Heavy Industries, Ltd. (Kawasaki Heavy Industries, Ltd, 2017). The fire-resistant hand (Tadakuma et al., 2020) grasped the fireball and ignited the torch. 3) The fireballs remained on fire and were subsequently extinguished by the DFF, a flying fire hose with improved reliability, as described below.

### 4.3 Mission of DFF

The detailed flow of the firefighting demonstration is as follows (Figure 2A):

• Take-off using water supplied by a fire engine as soon as possible.
• Approach the fireball in flight by the remote mobile base control (move for 4 m with a flight height over 1.5 m for appeal).
• An operator detects the fire point with normal and thermal imaging cameras mounted on the robot’s tip.
• The jets target the fireballs to extinguish the fire.
• After extinguishing the fire, the mobile base retreats, and the DFF lands.

The three fireballs (burning cloth with white gasoline) were placed at a height of approximately 0.6 m. Notably, fireballs cannot be easily extinguished and can reignite if they are not thoroughly extinguished.

Therefore, the requirements for developing the DFF are as follows:

1) The 4 m long robot can be remotely operated to extinguish fireballs that are 4 m in front of the head.
2) The robot should achieve a flying height of 1.5 m with the water supplied by a fire engine that is 20 m away (for safety) from the mobile base.
3) As this is a one-time-only demonstration, reliability should be improved to avoid failures.
4) Demonstration should be done in a short time. The duration for takeoff and landing should be shortened.

### 4.4 Robot design issues to achieve requirements

This paper mainly explains the following design issues to achieve the above requirements. 1) We installed a mobile base on the newly developed DFF (Section 5.1) to achieve remote control. 2) We redesigned the water channels (the sizes of nozzle outlets) for DFF to get enough thrust force to fly even with the 20 m long hose to the fire engine (Section 5.2). 3) To improve reliability, we newly developed nozzle units that extend the range of the rotating nozzles and developed waterproof covers for motors and microcomputers (Section 5.3). Because the risk of falling increases when the rotating nozzle reaches the limit of the movable range, the extension of the movable range contributes to increased reliability. 4) We redesigned the passive damping mechanism to gain better stability for this developed system, which contributes to dampening the oscillation caused by the takeoff movements (Section 5.4). Finally, we briefly explain the controller with a fire extinguishing mode (Section 5.5) and showed the demonstration results to prove that the above requirements are completely fulfilled (Section 6). We also discussed some lessons learned through the demonstrations (Section 7).

## 5 Developed DFF system

### 5.1 System overview

We developed a DFF system that satisfies the aforementioned four requirements. The DFF system consists of a 4 m long flying-hose-type firefighting robot, a mobile base to move the root of the hose, an operation computer to control the whole system, control boxes to manage commands and sensory data of DFF, and a fire engine to supply water. The system is illustrated in Figures 2A–C.

The robot was approximately 4 m long and equipped with nozzle units at the tip and middle at intervals of 2 m. The body of the DFF comprises a water-supply hose with an inner diameter of 25 mm and an outer corrugated tube with an outer diameter of 64 mm, both constructed from polypropylene (PP). The size of the internal hose was selected from the commercially available ones. The selected hose had inner and outer diameters of 25 and 33 mm, respectively. It could achieve the reaction force for levitation as discussed in section 5.2. Therefore, to accommodate this hose, wiring cables, and other essential components, the thinnest commercially available corrugated tube with an outer diameter of 64 mm and an inner diameter of 54 mm was utilized. We selected PP as the material because the tube can be made as flexible as possible with good heat resistance. In addition, we selected the corrugated tube to avoid buckling while achieving low stiffness in bending.

Microcomputer boards with an inertial measurement unit (IMU) were attached to the body by connecting elements at approximately 400 mm intervals. Each board calculates the posture of the IMU using the Madgwick filter at 100 Hz (Madgwick et al., 2011). This filter can compensate for the drift errors in roll and pitch postures by estimating the gravity direction using the acceleration sensor. For the drifts in the yaw angle, we applied a high-pass filter (a one-dimensional filter with a cut-off frequency of 0.1 Hz) to eliminate the accumulated errors, considering that the robot maintains a straight shape (the yaw angles of the distributed IMUs are approximately zero) in this demonstration. A damping mechanism was mounted at the root of the robot to suppress body vibrations (Yamaguchi et al., 2019).

The nozzle unit mounted at the tip and in the middle of the robot has four servo motors (Dynamixel MX-28AR, *ROBOTIS*) that drive the rotating nozzles, a microcomputer board that commands the rotation angles of the servo motors, and a camera (UR81X, *URVOLAX*), in addition to a main flow channel which branches out to four jet nozzles (Figure 6). The conventional controller (Eq. 1) and the nozzle angle optimization (Section 5.5) are realized in this microcomputer board based on the command $F_{i}^{n}$ from the operation computer (Figure 2C). We note that the velocity of the nozzle unit ${\dot{r}}_{i}$ is estimated from the posture and angular velocity data of all the IMUs mounted on the DFF (Ando et al., 2020; Ando et al., 2018), assuming that the mobile base stops. The head nozzle unit was equipped with a wire tensioning mechanism for damping mechanism and a thermo-camera (71201-0101, *FLIR*) to monitor the combustion process. The details of the nozzle unit are presented in Section 5.3.

The mobile base is a wheeled truck (HUSKY A200, *Clearpath Robotics Inc.*) and can be operated remotely with velocity inputs. It weighs 50 kg, has a payload of 75 kg, and can move at a maximum speed of 1.0 m/s. It can be operated remotely by connecting it to an operating computer in the operation center via Ethernet. It is equipped with a tower that consists primarily of an aluminum frame (*MISUMI*), and the height can be adjusted to a value approximately within the range of 1.5–2.3 m. The top of the tower is connected with the root of the DFF using 3D printer components; thereby, the root height can be adjusted to match that of the fire-extinguishing target. On the top of the tower, a fisheye camera (UR81X, *URVOLAX*) is installed to observe the robot’s shape in bird’s eye view. A flow meter (FD-R50, *Keyence*) and pressure gauge (GC61-174, *NAGANO KEIKI*) for the water channel of DFF are mounted at the rear of the tower.

The operation center contained the operation computer and two control and camera boxes. The control box is connected and communicates with all the microcomputer boards on the DFF to send commands to the nozzle unit and to get sensory data such as posture, angular velocity, and acceleration of each IMU, flow pressure, and flow rate with 100 Hz of communication frequency, in addition to supplying external power to the motor (15 V) and camera (12 V). The camera box has a video signal converter/distributor and a recording kit. It converts the camera image acquired by the nozzle unit for output to an operation monitor. The operation computer is connected to the control box and the mobile base, which can control the DFF system remotely.

The operator controls the DFF and mobile base at the operation center based on the images obtained by the camera mounted on the nozzle units and the mobile base in addition to the thermal image. In particular, the operator commands the constant force inputs $F_{i}^{n}$ for the two nozzle units and the velocity inputs for the mobile base (Figure 2C).

### 5.2 Flow channel design

First, to generate sufficient reaction force for the flight, the injection nozzle diameters of the nozzle units were determined by modeling and simulating the flow channel of the DFF based on the previous study (Ando et al., 2020). Compared with (Ando et al., 2020), we constructed a more detailed model to estimate the reaction force.

#### 5.2.1 Model

**FIGURE 3:**
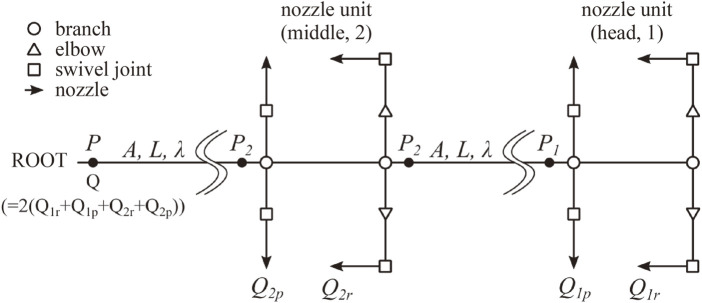
Flow channel model for flow simulation.

The flow channel of the DFF was modeled, as shown in Figure 3. The flow channel extends from the root (the mobile base) to the middle nozzle unit via a 2 m hose and from the middle nozzle unit to the head nozzle unit again via a 2 m hose. The water branches out to four nozzle outlets open to the atmosphere at each nozzle unit, as described in Section 5.3. In the model, we describe the friction factor of the main hose as *λ*, the pressure drop coefficient for water channel instruments as *ξ*_*i*_, the cross-sectional area of the hose as *A*, the hose length as *L*, the pressure before the head nozzle unit as *P*_1_, the pressure before and after the middle nozzle unit as *P*_2_, and the total flow rate as *Q*. The flow rates of the rotating nozzle outlets on nozzle unit *i* are described as *Q*_*ip*_ and *Q*_*ir*_, where *p* and *r* represent the pitch and roll rotating nozzles, respectively. Pressure loss is assumed to occur at the hose (coefficient: *λ*), branch (coefficient: *ξ*_*B*_), elbow pipe (coefficient: *ξ*_*el*_), swivel joint (coefficient: *ξ*_*SW*_), nozzle (coefficient: *ξ*_*nzls*_), and joint point between the outlet of middle nozzle unit and inlet of the front main hose (coefficient: *ξ*_*c*_). We assume that the main water channel of each nozzle unit does not cause energy loss because their lengths are small. The cross-sectional areas for the branch, elbow pipe, swivel joint, nozzle, and main channel inside the nozzle unit are described as *A*_*B*_, *A*_*el*_, *A*_*SW*_, *A*_*nzls*_, and *A*_*c*_, respectively. The values of those physical parameters are listed in Table 1. We note that the friction factor and pressure drop coefficients *λ*, *ξ*_*c*_, *ξ*_*B*_, *ξ*_*el*_, *ξ*_*SW*_, and *ξ*_*nzls*_ were estimated experimentally in advance.

**TABLE 1:** Parameters of the flow simulation.

Elements	Value	Elements	Value
*λ*	0.016	A [*mm* ^2^]	490.87
*ξ*_*c*_	1.0224	*A*_*c*_ [*mm* ^2^]	415.48
*ξ*_*B*_	0	*A*_*B*_ [*mm* ^2^]	95.03
*ξ*_*el*_	1.547	*A*_*el*_ [*mm* ^2^]	95.03
*ξ*_*SW*_	2.5635	*A*_*SW*_ [*mm* ^2^]	78.54
*ξ*_*nzls*_	0.1327	L [m]	2.0

From Bernoulli’s theorem for an inner nozzle and hose, we can derive the following equations:

$$\frac{P_{2}}{\rho }-\frac{P_{1}}{\rho }=\frac{\lambda }{2}{\left(\frac{2\left({Q_{1}}_{r}+{Q_{2}}_{p}\right)}{A}\right)}^{2}\frac{L}{2\sqrt{A/\pi }}+\frac{1}{2}\xi_{c}{\left(\frac{2\left({Q_{1}}_{r}+{Q_{2}}_{p}\right)}{A_{c}}\right)}^{2}$$ (2)

$$\frac{P}{\rho }-\frac{P_{2}}{\rho }=\frac{\lambda }{2}{\left(\frac{2\left({Q_{1}}_{r}+{Q_{1}}_{p}+{Q_{2}}_{r}+{Q_{2}}_{p}\right)}{A}\right)}^{2}\frac{L}{2\sqrt{A/\pi }}$$ (3)

$$\begin{array}{ll} \frac{P_{1}}{\rho }+\frac{1}{2}{\left(\frac{2{Q_{1}}_{r}}{A_{c}}\right)}^{2} & =\frac{P_{a}}{\rho }\;+\;\frac{1}{2}{\left(\frac{{Q_{1}}_{r}}{{A_{1}}_{r}}\right)}^{2}\;+\;\frac{1}{2}\xi_{B}{\left(\frac{{Q_{1}}_{r}}{A_{B}}\right)}^{2}\;+\;\frac{1}{2}\xi_{el}{\left(\frac{{Q_{1}}_{r}}{A_{el}}\right)}^{2}\\ & \;+\;\frac{1}{2}\xi_{SW}{\left(\frac{{Q_{1}}_{r}}{A_{SW}}\right)}^{2}\;+\;\frac{1}{2}\xi_{\mathrm{nzls}}{\left(\frac{{Q_{1}}_{r}}{A_{\mathrm{nzls}}}\right)}^{2} \end{array}$$ (4)

$$\begin{array}{ll} \frac{P_{1}}{\rho }+\frac{1}{2}{\left(\frac{2\left({Q_{1}}_{r}+{Q_{1}}_{p}\right)}{A}\right)}^{2} & =\frac{P_{a}}{\rho }+\frac{1}{2}{\left(\frac{{Q_{1}}_{p}}{{A_{1}}_{p}}\right)}^{2}+\frac{1}{2}\xi_{B}{\left(\frac{{Q_{1}}_{p}}{A_{B}}\right)}^{2}\\ & \;+\frac{1}{2}\xi_{SW}{\left(\frac{{Q_{1}}_{p}}{A_{SW}}\right)}^{2}+\frac{1}{2}\xi_{\mathrm{nzls}}{\left(\frac{{Q_{1}}_{p}}{A_{\mathrm{nzls}}}\right)}^{2} \end{array}$$ (5)

$$\begin{array}{ll} & \frac{P_{2}}{\rho }+\frac{1}{2}{\left(\frac{2\left({Q_{1}}_{r}+{Q_{1}}_{p}+{Q_{2}}_{r}\right)}{A_{c}}\right)}^{2}\\ & =\frac{P_{a}}{\rho }+\frac{1}{2}{\left(\frac{{Q_{2}}_{r}}{{A_{2}}_{r}}\right)}^{2}+\frac{1}{2}\xi_{B}{\left(\frac{2\left({Q_{1}}_{r}+{Q_{1}}_{p}+{Q_{2}}_{r}\right)}{A_{B}}\right)}^{2}+\frac{1}{2}\xi_{el}{\left(\frac{{Q_{2}}_{r}}{A_{el}}\right)}^{2}\\ & \;+\frac{1}{2}\xi_{SW}{\left(\frac{{Q_{2}}_{r}}{A_{SW}}\right)}^{2}+\frac{1}{2}\xi_{\mathrm{nzls}}{\left(\frac{{Q_{2}}_{r}}{A_{\mathrm{nzls}}}\right)}^{2} \end{array}$$ (6)

$$\begin{array}{ll} \frac{P_{2}}{\rho }+\frac{1}{2}{\left(\frac{Q}{A}\right)}^{2} & =\frac{P_{a}}{\rho }+\frac{1}{2}{\left(\frac{{Q_{2}}_{p}}{{A_{2}}_{p}}\right)}^{2}+\frac{1}{2}\xi_{B}{\left(\frac{Q}{A_{B}}\right)}^{2}+\frac{1}{2}\xi_{SW}{\left(\frac{{Q_{2}}_{p}}{A_{SW}}\right)}^{2}\\ & \;+\frac{1}{2}\xi_{\mathrm{nzls}}{\left(\frac{{Q_{2}}_{p}}{A_{\mathrm{nzls}}}\right)}^{2} \end{array}$$ (7)

where Equations 2, 3 are equations of energy conservation for two hoses of length *L*, and Equations 4–7 are those for the channels from the nozzle outlets (head roll nozzle, head pitch nozzle, middle roll nozzle, middle pitch nozzle) to the closest branches, respectively. *P*_*a*_ is the atmospheric pressure and is set as 0.1 MPa. If we set the pressure of root *P*, the six variables *P*_1_, *P*_2_, ${Q_{1}}_{r}$ , ${Q_{1}}_{p}$ , ${Q_{2}}_{r}$ , and ${Q_{2}}_{p}$ can be numerically solved using the above six equations. In addition, the injection reaction forces are obtained using the formula $F_{i}=\rho Q_{i}^{2}/A_{\mathrm{nzls}}$ for *i* ∈ {1*p*, 1*r*, 2*p*, 2*r*}, where *ρ* is the water density 1,000 kg/m^3^.

#### 5.2.2 Results of numerical calculation

**FIGURE 4:**
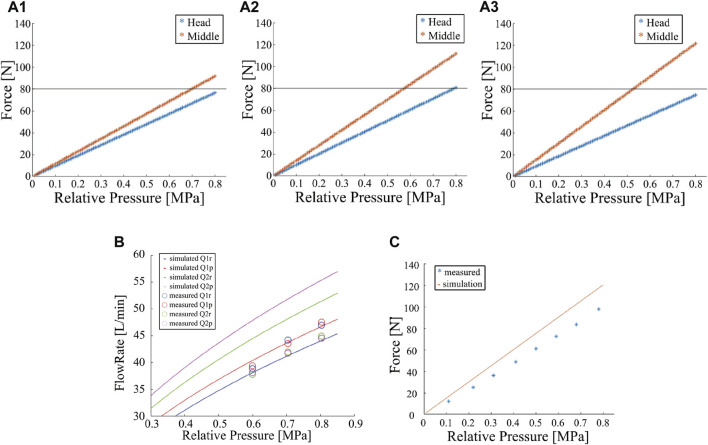
Results of the flow channel design simulation. **(A)** Simulated results of the sum of the reaction forces at nozzle units against the root pressure for various nozzle outlets (A1:*ϕ*5, A2:*ϕ*6, A3:*ϕ*7 mm). **(B)** Simulated and experimental results of the flow rate of each nozzle against the root pressure. **(C)** Simulated and experimental results of the sum of the reaction forces at a nozzle unit against the root pressure.

We solve Equations 2–7 using the *fsolve* function in *MATLAB*. Figures 4A1–A3 show the summation of the reaction forces from four nozzles on each nozzle unit against the root pressure *P* for *A*_*nzls*_ = 19.6, 28.3, and 38.5 mm^2^ (diameters of 5, 6, and 7 mm), respectively. Based on the preliminary simulation result, we aim to realize a total reaction force of the head and middle nozzle units over 80 N. In addition, we estimated that the fire engine can generate a root pressure *P* up to a relative pressure of 0.9 MPa. Thus, we attempted to realize an 80 N force at a relative pressure of 0.8 MPa with an extra margin. The results show that a nozzle diameter of 5 mm (Figure 4A1) does not provide sufficient reaction force because of insufficient flow rate, whereas a nozzle diameter of 7 mm (Figure 4A3) increases the flow rate but does not provide a reaction force at the head nozzle unit because a large amount of water is expelled from the middle nozzle unit. The case with a nozzle diameter of 6 mm fulfills the requirement. Therefore, a nozzle diameter of 6 mm was selected for this study.

#### 5.2.3 Comparison with the experimental data

We also conducted experiments with the actual robot to validate the results of the flow channel model. We prepared the developed 4 m DFF with the discussed flow channel and supplied water from the root using a fire pump (VC72PROIII Limited, *TOHATSU*) while measuring the flow rate and pressure at the root. We fixed the pump pressure for each experimental trial and gathered water from four nozzles (the roll and pitch nozzles on the head and middle nozzle units) in four tanks for 10 s to measure *Q*_1*p*,1*r*,2*p*,2*r*_. We repeated this trial three times for various root pump pressures (*P*) of 0.6, 0.7, and 0.8 MPa.

The results are shown in Figure 4B, along with the model simulation results. The line represents the simulation results, and the circles represent the mean of the measured flow rates. The color represents the type of nozzle outlets. The results show that the flow rates from the head nozzle unit correspond well between the simulation and experiments: the maximum error is approximately 10%. The flow rate from the middle nozzle is lower in the experiment; The maximum error is approximately 20%.

Further, the injection reaction force was measured and compared between the experiment and simulation with a 2 m long DFF which only has the head nozzle unit. We could not measure the forces with 4 m DFF because the robot was too long to allocate the force sensors. In the experiment, the water was supplied from a fire pump and was connected to the DFF via a flow meter and pressure gauge. The nozzle unit was fixed to a six-axis force sensor. All four nozzles were oriented vertically downward, the pressure *P* varied from 0.1 to 0.8 MPa, and the injection reaction force was measured at eight points every 0.1 MPa. As the simulation model, we constructed a single-nozzle unit model with a 2 m hose, and the equations were reformulated and simulated using the same parameters.

The measurement results of the injection reaction force are shown in Figure 4C, along with the simulation results. Although the tendency corresponds well between the simulation and experiments, the reaction force estimated from the simulation was approximately 20% greater than the measured reaction force.

#### 5.2.4 Discussion on observed errors

The results of these two experiments indicated a maximum difference of approximately 20% between the experiment and simulation. One possible reason for this difference is the estimated error of the pressure loss coefficients. In this model, we estimated the coefficients individually in a simple experimental situation. However, actual flow channels are complex, and these elements, which are assumed to have pressure loss, are connected together. Therefore, the coefficients may change from the estimated values when integrated. To eliminate this discrepancy, the estimation method should be changed in the future.

Despite the above discrepancy, fortunately, the flow rates of the head nozzle unit are almost underestimated in the simulation in Figure 4B. Because only the head nozzle’s total force is near the targeted force threshold of 80 N and the middle nozzle generates much larger force than the threshold (1.5 times), as shown in Figure 4A2, we think our designed nozzle diameter can fulfill the threshold even in the actual DFF robot.

### 5.3 Development of nozzle unit

As mentioned in Section 3, the conventional DFF has limited injection ranges of the rotating nozzles (±60°), and the servo motors suffer from water leakage. In addition, the nozzle units are made of resin fabricated by a 3D printer, which is vulnerable to impact.

In this section, we introduce a developed nozzle unit made from aluminum to improve the strength, and to expand the jetting range, as well as ensure waterproofing of the motors and microcomputers. First, a metalized main channel for nozzle unit is introduced. Then, a waterproof case is introduced to prevent motor failure due to water leakage. Then we introduce a small microcomputer board and its waterproof case to improve the communication performance. Finally, we introduce a metalized nozzle unit that incorporates swivel joints to expand the injection range.

#### 5.3.1 Metalized primary water channel

The primary channel is metalized to improve its strength. The conventional primary channel is fabricated using a 3D printer, causing water leakage at the connection point with the hose, in addition to limited strength. However, the metallization of the primary channel with the current design would render it heavier. Therefore, strength calculations were performed to reduce the wall thickness.

Assuming that the primary channel is a thick-walled cylinder, a wall thickness that is sufficiently strong to withstand internal pressure was determined. Generally, a cylinder subjected to internal pressure is subjected to three types of stresses: circumferential, radial, and longitudinal. According to the theory of the maximum principal stress, the circumferential stress on the inner surface of the cylinder was used as the design stress. Assuming that the outer diameter is *D*, wall thickness is *t*, and internal pressure is *P*, the designed stress *σ*_*t*_ is given as follows:

$$\sigma_{t}=\frac{\frac{1}{2}{\left(\frac{D}{t}\right)}^{2}-\frac{D}{t}+1}{\frac{D}{t}-1}P$$ (8)

If the inner radius is *r*, *D* = 2*r* + 2*t*. Substituting and rearranging yields the following quadratic equation: The wall thickness *t* is a solution to this quadratic equation.

$$\left(\sigma_{t}-P\right)t^{2}+2r\left(\sigma_{t}-P\right)-2r^{2}P=0$$ (9)

The inside diameter *r* and applied pressure *P* are assumed to be *r* = 11.5 mm and *p* = 1 MPa. *P* is approximately 1 MPa, which is similar to the root pump pressure of the DFF. The material is assumed to be lightweight and high-strength duralumin (A7075), and the design pressure *σ*_*t*_ is set as the yield strength of A7075 (505 MPa) divided by a safety factor of 7. By substituting *σ*_*t*_ into (Eq. 9), the wall thickness *t* is obtained as 0.16 mm.

**FIGURE 5:**
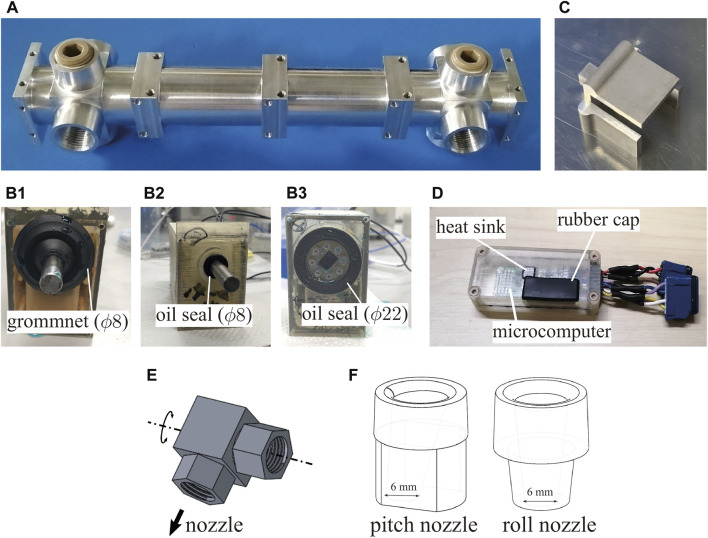
Photos or CADs of the developed nozzle unit parts. **(A)** Photo of the primary channel. **(B)** Proposed waterproof cases of the servomotor. **(C)** Part transmitting torque of the motor. **(D)** Waterproof case of the inertial measurement unit microcomputer. **(E)** CAD of swivel joint. **(F)** CAD of nozzle.

The primary channel shown in Figure 5A is fabricated using the results of the strength calculations. The total length of the primary channel is 232 mm. In addition to the inlet and outlet, the primary channel has eight branches, including a spare. The wall thickness was approximately 3 mm, sufficiently thicker than the calculated value of 0.16 mm to allow for a 3/4-inch tapered thread at the inlet/outlet and machining of the threads. Each branch of the nozzle also had a 3/8 inch tapered thread.

#### 5.3.2 Waterproofed motors

We developed a waterproof case to protect the motor from water by waterproofing the motor’s output shaft and the hole through which the cable passed. The motor used was Dynamixel MX-28AR (*ROBOTIS*), which was not waterproofed. The waterproof case fabricated using a 3D printer comprised an open case body and a lid. Waterproof and dustproof grommets (T-1475716-P50; *Sugatsune Industries*) were used to waterproof the cables. The cables were bundled with heat-shrinkable tubes, and gaps were filled with adhesive.

We developed the following three waterproofing structures for the output shaft (Figures 5B1–B3) and checked the waterproofing ability. For two structures (B1 and B2), the rotation torque was transferred by the output shaft made of A7075, whose diameter was approximately 8 mm, according to strength calculations. B1 and B2 use the grommet (*ϕ*8 mm) and oil seal (*ϕ*8 mm) to provide protection from water leakage, respectively. These are normal waterproofing techniques. In contrast, in the third case, B3, the diameter of the output shaft was larger than that in the other cases, and the shaft has a drilled squared hole. Although this design increases the area of sealing surfaces due to the larger diameter, this can reduce the shaft length because the axial length of the sealing part can also be used for the length of the squared hole to transmit the torque to another instrument. Case B3 uses the oil seal (*ϕ*22 mm) for sealing.

We conducted a water leakage test. In the test, each case rotates the shaft for 30 min inside a water depth of 50 mm. The experimental results showed that no shaft leakage was observed in any of the three proposed waterproofing methods. We decided to select the B3 case for installation based on the advantage of reducing the shaft length.

**FIGURE 6:**
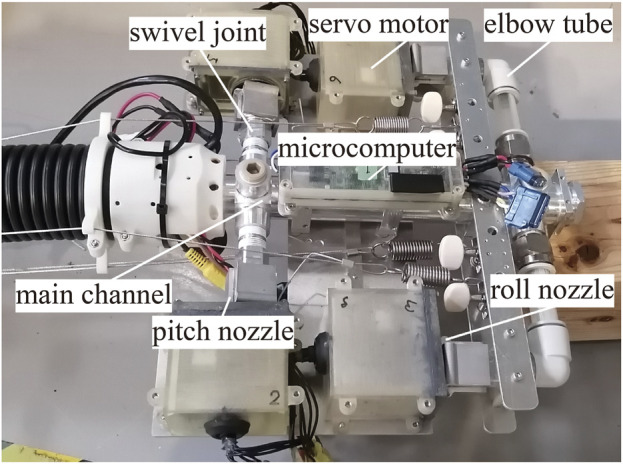
Photo of the head nozzle unit.

Based on the shaft structure of B3, we designed a U-shaped connecting instrument (Figure 5C), which could transmit torque to the shaft through the squared convex cube. This U-shaped instrument can cover the swivel joints (Figure 5E) as Figure 6. The details are explained in Section 5.3.4.

#### 5.3.3 Developed and waterproofed a small microcomputer board

We developed a small microcomputer board and a case to protect the board to improve communication performance. Thus far, microcontrollers used in DFFs were not for long robots, such as a DFF, but for general robots, resulting in frequent communication errors. To address this issue, we first developed a small microcomputer board equipped with an IMU. These boards can connect serially over the length of 10 m and communicate with each other at 20 Mbps.

Then, we developed a waterproof case for the board, as shown in Figure 5D. The waterproof case consists of a lower box and an upper cover. The board is fixed on the lower box. The cables for communication are located between the cover and box, and the cables are sealed with the box and cover with adhesive. These parts were printed using an optical 3D printer. In addition, the cover had a hole, which was covered with a rubber cap, to program the microcomputer from the outside. The cover also has a heat-dissipating column made of an aluminum square pole (7 mm × 7 mm) bonded to the microcontroller with an electric heating sheet.

#### 5.3.4 Nozzle unit with extended range of jetting directions

Figure 6 shows the developed nozzle unit. The nozzle has four subchannels connected to injection nozzles, which are branched from the primary flow channel, as described above. All the subchannels were connected to the injection nozzles via swivel joints at their ends. The subchannels branched at the root side rotated in the pitch direction, whereas those branched at the head side bent the 90° channel to achieve a roll direction. The head side was also branched downward and was connected to a mist nozzle to protect the nozzle unit from heat.

To realize the large injection direction range, we installed a specially designed swivel joint that can rotate for 360° around an axis. Figure 5E illustrates the swivel joint used. The swivel joint is made of metal and has high mechanical strength. One side of the swivel is connected by the injection nozzle (Figure 5F), and the other side is connected to the subchannel of the nozzle unit. To rotate the swivel joint, the swivel joint is covered by the U-shaped instrument connected to the motor case (Figure 6). This mechanism can rotate the swivel joint for 360° around an axis. Considering that the nozzle unit has a cover to protect it, the possible range of injection angles is approximately ±90° for the pitch nozzle and ±80° for the roll nozzle, which is almost 1.5 times larger than the conventional nozzle unit.

We note that the U-shaped instrument does not constrain the horizontal movement of the swivel joint, which means that the swivel joint can slide toward the opened end of the U-shaped instrument. In addition, the motor shaft with the squared hole (not a cubic hole) also allowed the U-shaped instrument to move along the opened directions of the square. These structures act like a coupling, which permits some degree of misalignment while the rotating torque can be transmitted.

The injection nozzle is made of metal, as shown in Figure 5F, as is the swivel joint. The inlet and outlet diameters were set to 10 and 6 mm, respectively, to narrow the flow channel to the appropriate diameters, as described above. The inlet was filleted to smoothen the flow channel. The pitch nozzle was tilted outward at an angle of 10° to prevent water injection at the rear from hitting the body and holding it down.

### 5.4 Development of passive damping mechanism

In this section, to realize better system stability, we installed a passive damping mechanism (Yamaguchi et al., 2019) on the newly developed DFF. We applied almost the same procedure of (Yamaguchi et al., 2019) to the newly developed DFF. In the implementation, the specification of the damper coefficient of the mechanism is crucial to minimize the convergence time of the body vibration. Thus, first, we designed the planar model of the DFF and identified its parameters. Then, the optimum damper coefficients were selected through dynamic simulation, and we implemented the mechanism on the DFF.

#### 5.4.1 Overview of passive damping mechanism

**FIGURE 7:**
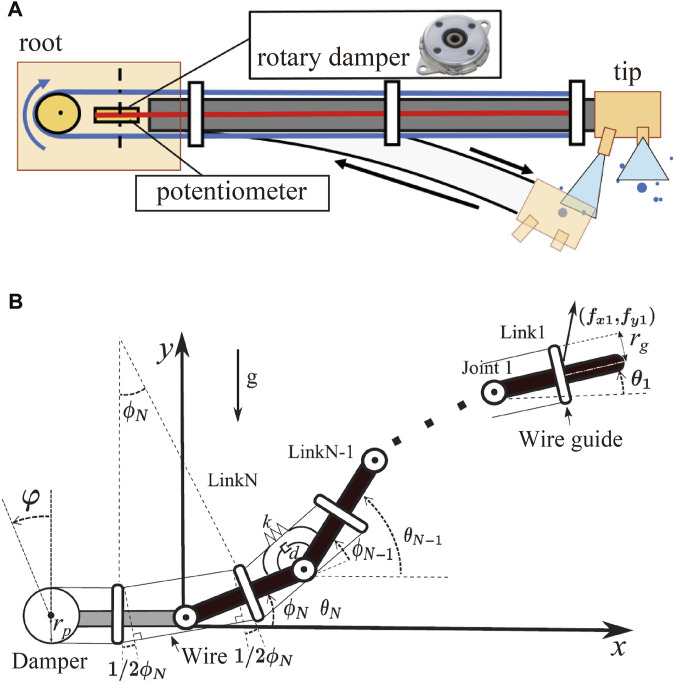
**(A)** Overview of the mechanism of damping vibration. **(B)** Sagittal model of DFF with the damping mechanism.

The passive damping mechanism comprises a wire crawling along the body and folding back at the root of the DFF, as shown in Figure 7A. A rotary damper, which is a rotating damper, is installed at the folded part. This mechanism provides damper performance when the body deforms. Considering the case in which the body deforms downward, as shown in Figure 7A, the lengths of the upper and lower wires change. This causes the lower wire to move to the upper side, and the rotary damper rotates with this movement to perform damping. This mechanism is suitable for flying long robots, such as a DFF, because it does not require an actuator and is lightweight and simple for vibration control.

#### 5.4.2 Planar model of DFF

The DFF is approximated as a model comprising *N* rigid links (weight: *m*_*i*_ > 0, moment of inertia: *I*_*i*_ > 0, length: *l* > 0) and rotating joints (spring constant: *k*, damper constant: *d*) in the *xy* plane, as shown in Figure 7B (Yamaguchi et al., 2019). Each link is numbered 1, … , *N*, starting from the head link. Let *θ*_*i*_ be the pitch angle of link *i* (in the general coordinate system of the model), *ϕ*_*i*_ be the joint angle, (*x*_*i*_, *y*_*i*_) be the position of the center of gravity, and ( $f_{x_{i}}$ , $f_{y_{i}}$ ) be the external force on the center of gravity. This external force represents the net force that the nozzle unit generates. The gravitational acceleration is *g*, the width of the wire guide is 2*r*_*g*_, the pulley radius of the rotary damper is *r*_*p*_, the rotation angle of the rotary damper is *ϕ*, and the damper coefficient of the rotary damper is *c*.

Further, to simplify the derivation, vectors whose elements are *x*_*i*_, *y*_*i*_, *θ*_*i*_, *m*_*i*_, *ϕ*_*i*_, $f_{x_{i}}$ , and $f_{y_{i}}$ (i = 1, … , N) are ***x***, ***y***, ***θ***, ***ϕ***, ***f***_*x*_, and ***f***_*y*_ ∈ $R^{N\times 1}$ . In this case, the kinetic energy *T* and potential energy *V* are given by (10) and 11, respectively.

$$T=\frac{1}{2}\left({\dot{x}}^{T}M\dot{x}+{\dot{y}}^{T}M\dot{y}+{\dot{\theta }}^{T}J\dot{\theta }\right)$$ (10)

$$V=\frac{1}{2}k{\left(R\theta \right)}^{T}R\theta +lgm^{T}A\sin\theta$$ (11)

where $\dot{x}$ , *A*, *S*_*θ*_, *C*_*θ*_ and *R* are given by:

$$\begin{array}{ccc} & \dot{x}=-lAS_{\theta }\dot{\theta },\;\;\dot{y}=lAC_{\theta }\dot{\theta } & \\ & \;A=\left[\begin{array}{cccc} \frac{1}{2} & 1 & \cdots & 1\\ 0 & \frac{1}{2} & \cdots & 1\\ \vdots & \vdots & \ddots & \vdots \\ 0 & 0 & \cdots & \frac{1}{2} \end{array}\right] & \\ & S_{\theta }=diag\left(\sin\theta_{1},\ldots ,\sin\theta_{N}\right),\;\;C_{\theta }=diag\left(\cos\theta_{1},\ldots ,\cos\theta_{N}\right) & \\ & M=diag\left(m\right),\;\;J=diag\left(I\right),\;\;\sin\theta ={\left[\sin\theta_{1},\ldots ,\theta_{N}\right]}^{T} & \\ & R=\left[\begin{array}{ccccc} 1 & -1 & 0 & \cdots & 0\\ 0 & 1 & -1 & \cdots & 0\\ 0 & 0 & 1 & \cdots & 0\\ \vdots & \vdots & \vdots & \ddots & \vdots \\ 0 & 0 & 0 & \cdots & 1 \end{array}\right] & \end{array}$$

*R* is an *N* × *N* matrix that satisfies *ϕ* = *Rθ*. The dissipative function *U* of the model can be expressed as (Eq. 12).

$$\begin{array}{ll} U & =\frac{1}{2}d{\dot{\phi }}^{T}\dot{\phi }+\frac{1}{2}c{\dot{\varphi }}^{2}\\ & =\frac{1}{2}d{\dot{\theta }}^{T}R^{T}R\dot{\theta }+\frac{1}{2}c{\left(\frac{r_{g}}{r_{p}}\right)}^{2}{\dot{\theta }}^{t}bb^{T}\dot{\theta } \end{array}$$ (12)

$$b=\left[\begin{array}{c} \cos\frac{1}{2}\left(\theta_{1}-\theta_{2}\right)\\ \cos\frac{1}{2}\left(\theta_{2}-\theta_{3}\right)-\cos\frac{1}{2}\left(\theta_{1}-\theta_{2}\right)\\ \vdots \\ \cos\frac{1}{2}\left(\theta_{N-1}-\theta_{N}\right)-\cos\frac{1}{2}\left(\theta_{N-2}-\theta_{N-1}\right)\\ \cos\frac{1}{2}\theta_{N}-\cos\frac{1}{2}\left(\theta_{N-1}-\theta_{N}\right) \end{array}\right]$$

Using the above-mentioned results, the Lagrangian *L* = *T* − *V*, and dissipative function *U*, we obtain the equation of motion (Eq. 13). This equation can be revised as (Eq. 14).

$$\frac{d}{dt}\frac{\partial L}{\partial \dot{\theta }}-\frac{\partial L}{\partial \theta }+\frac{\partial U}{\partial \dot{\theta }}=-lf_{x}^{T}AS_{\theta }+lf_{y}^{T}AC_{\theta }$$ (13)

$$\begin{array}{ll} & H\ddot{\theta }+D\dot{\theta }+kR^{T}R\theta +dR^{T}R\dot{\theta }+c{\left(\frac{r_{g}}{r_{p}}\right)}^{2}bb^{T}\dot{\theta }+lgC_{\theta }A^{T}m\\ & =-lS_{\theta }A^{T}f_{x}+lC_{\theta }A^{T}f_{y} \end{array}$$ (14)

where *H* and *D* are represented as

$$\begin{array}{ll} H & =S_{\theta }\bar{M}S_{\theta }+C_{\theta }\bar{M}C_{\theta }+J\\ D & =\left(S_{\theta }\bar{M}C_{\theta }+C_{\theta }\bar{M}S_{\theta }\right)diag\left(\dot{\theta }\right)\\ \bar{M} & =l^{2}A^{T}MA \end{array}$$

The mass, length, and time were then non-dimensionalized using *m*, *l*, and $\sqrt{l/g}$ , respectively. The non-dimensionalized values are denoted by *. The equation of motion after non-dimensionalization is given by (Eq. 15).

$$\begin{array}{ll} & H^{*}{\ddot{\theta }}^{*}+D^{*}{\dot{\theta }}^{*}+k^{*}R^{T}R\theta^{*}+d^{*}R^{T}R{\dot{\theta }}^{*}+c^{*}{\left(\frac{r_{g}^{*}}{r_{p}^{*}}\right)}^{2}b^{*}{b^{*}}^{T}{\dot{\theta }}^{*}+C_{\theta }A^{T}m^{*}\\ & =-S_{\theta }A^{T}f_{x}^{*}+C_{\theta }A^{T}f_{y}^{*} \end{array}$$ (15)

$$\begin{array}{ll} H^{*} & =S_{\theta }{\bar{M}}^{*}S_{\theta }+C_{\theta }{\bar{M}}^{*}C_{\theta }+J^{*}\\ D^{*} & =\left(S_{\theta }{\bar{M}}^{*}C_{\theta }+C_{\theta }{\bar{M}}^{*}S_{\theta }\right)diag\left({\dot{\theta }}^{*}\right)\\ {\bar{M}}^{*} & =A^{T}M^{*}A \end{array}$$

#### 5.4.3 Parameter identification

**FIGURE 8:**
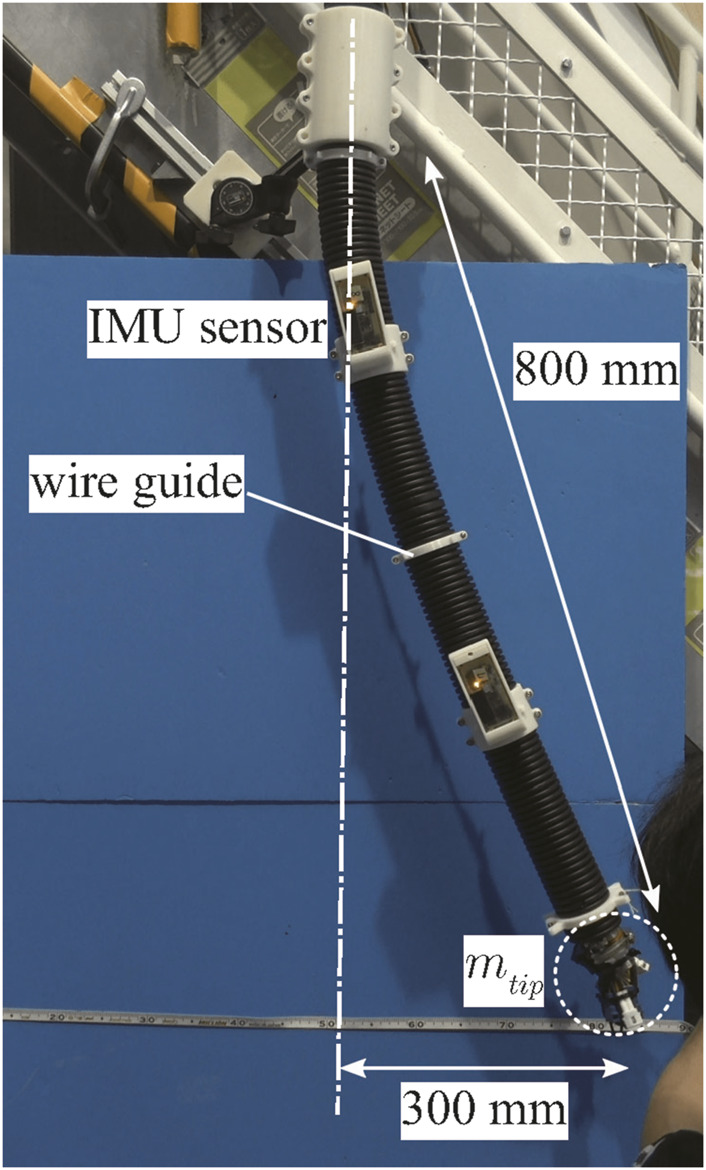
Experimental setup to estimate k* and d*.

To identify the spring constant k* and damper constant d* of joints in the model, we compared the vibration movement of a part of the DFF between the simulation and the experiment. In the experiment, a part of the DFF body (800 mm) was suspended vertically by fixing the root of the body in the direction of gravity as Figure 8. The tip of the body was initially displaced by approximately 300 mm horizontally from the root and released. The time response of the posture where the front side IMU is attached was measured at 100 Hz by the front side IMU. We repeated this experiment five times. For the simulation, a rigid four-link model was used. The parameters were set as listed in Table 2 based on the measurements. The moment of inertia *I*_*i*_ was calculated assuming a uniform cylinder with a weight of *m*_*i*_ and a diameter of 64 mm (equivalent to the diameter of the corrugated tube body). We solved the equation of motion (Eq. 14) numerically using the *ode45* function in MATLAB using *g* = 9.81 m/s^2^.

**TABLE 2:** Parameters for experiment to estimate k* and d*.

Element	Value	Element	Value	Element	Value
*N*	4	*l* [m]	0.2	*m* [kg]	0.1953
*m*_1_ [kg]	0.2667	$m_{1}^{*}$	1.3655	$I_{1}^{*}$	0.0369
*m*_2_ [kg]	0.3001	$m_{2}^{*}$	1.5363	$I_{2}^{*}$	0.1367
*m*_3_ [kg]	0.2044	$m_{3}^{*}$	1.0464	$I_{3}^{*}$	0.0931
*m*_4_ [kg]	0.3001	$m_{4}^{*}$	1.5363	$I_{4}^{*}$	0.1367

**FIGURE 9:**
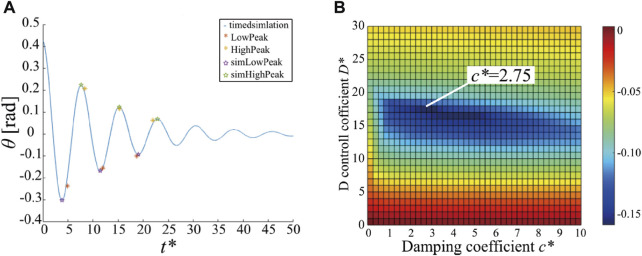
Results of parameter estimation **(A)** and stability improvement with damping mechanism **(B)**. **(A)** The extreme high and low values of the oscillation measured from the front side IMU (red and yellow asterisks) and those generated from the simulation using estimated joint stiffness and damping parameters (purple and green stars with blue line). **(B)** Maximum real part of the eigenvalues of the Jacobian matrix *versus* the D gain of the controller s* and the coefficient of damping mechanism c*.

One of the experimental results is presented in Figure 9A. The red and yellow asterisks represent the high and low extreme values of the measured oscillation of the front side IMU, respectively. We plotted only six extreme values. Through the simulation, we estimated k* and d* by fitting them to the six extreme values using the least squares method. The blue line represents the time response of the posture of link 2 with the estimated k* and d*. The purple and green stars represent the high and low extreme values of the simulation results. This estimation procedure was performed five times, and then, we obtained an average of k* = 13.41 and d* = 6.603. (In dimensional quantities, *k* = 5.139 N/rad and *d* = 0.3613 N/(rad/s)).

#### 5.4.4 Selection of damping coefficient of the mechanism

We conducted the simulation and stability analysis with a 4 m DFF model to specify the damping coefficient of the mechanism *c*. The simulation parameters are listed in Table 3. The length of the DFF was 4 m, the number of links N was 19, and links 1 and 11 were nozzle units approximated as rectangular links (400 mm × 400 mm × 100 mm) with a mass of approximately 2.7 kg.

**TABLE 3:** Parameters for simulations.

Element	Value	Element	Value	Element	Value
*m* [kg]	0.2935	*l* [m]	0.2	*g* [m/s^2^]	9.81
$m_{1,11}^{*}$	9.4175	$m_{2,4,\ldots ,18}^{*}$	1.0309	$m_{3,5,7,9,13,15,17,19}^{*}$	1.3569
$I_{1,11}^{*}$	3.3354	$I_{2,4,\ldots ,18}^{*}$	0.0917	$I_{3,5,7,9,13,15,17,19}^{*}$	0.1207
*N*	19	k*	8.9244	$r_{g}^{*}$	0.175
		d*	4.3943	$r_{p}^{*}$	0.18

The forces acting on the nozzle units were controlled by the following equations based on (Eq. 1).

$${\left[f_{x1}\;f_{y1}\right]}^{T}={\left[F_{x1}\;F_{y1}\right]}^{T}-s\left[{\dot{x}}_{1}\;{\dot{y}}_{1}\right],\;\;{\left[f_{\mathrm{x11}}\;f_{\mathrm{y11}}\right]}^{T}={\left[F_{\mathrm{x11}}\;F_{\mathrm{y11}}\right]}^{T}-s\left[{\dot{x}}_{11}\;{\dot{y}}_{11}\right]$$ (16)

where *s* is the damping gain of the controller, (*F*_*x*1_, *F*_*y*1_) = (50.9, 50.9) N and (*F*_*x11*_, *F*_*y11*_) = (83.1, 48) N.

A linear stability analysis with a Jacobian matrix was used to evaluate damping performance. The Jacobian matrix is defined according to the equation of motion (14). Let $z={\left[\theta^{T}{\dot{\theta }}^{T}\right]}^{T}$ be set as the state vector of the system. Then, (Eq. 14) can be written as expressed as

$$\frac{dz}{dt}=g\;\left(z\right)$$ (17)

where ***g*** (***z***) is a non-linear vector function. The Jacobian matrix *J* can be defined at the equilibrium point ***z*** * (***g*** (***z*** *) = ***0***) as follows:

$$J={\left.\frac{\partial g}{\partial z}\right|}_{z=z^{*}}$$ (18)

The eigenvalues of the Jacobian matrix specify the linear stability of the system. The system is linearly stable when the real parts of all eigenvalues of *J* are less than zero; and the maximum real part of all eigenvalues determines the damping performance.

In the simulation, linear stability analysis of the equilibrium point was conducted by changing the control parameter *s* and rotary damping parameter *c*. For the stability evaluation, the maximum real part of the eigenvalues of the Jacobian matrix was used. Figure 9B shows a color map of the maximum real part of the eigenvalues against *s* and *c*. The colder the color, the smaller the maximum value of the eigenvalue, implying that the oscillations converge faster. Notably, vibration is the most stable at c* = 2.75 (*c* ≈ 0.23*Nm*/(*rad*/*s*)).

#### 5.4.5 Implementation of the mechanism to the DFF

**FIGURE 10:**
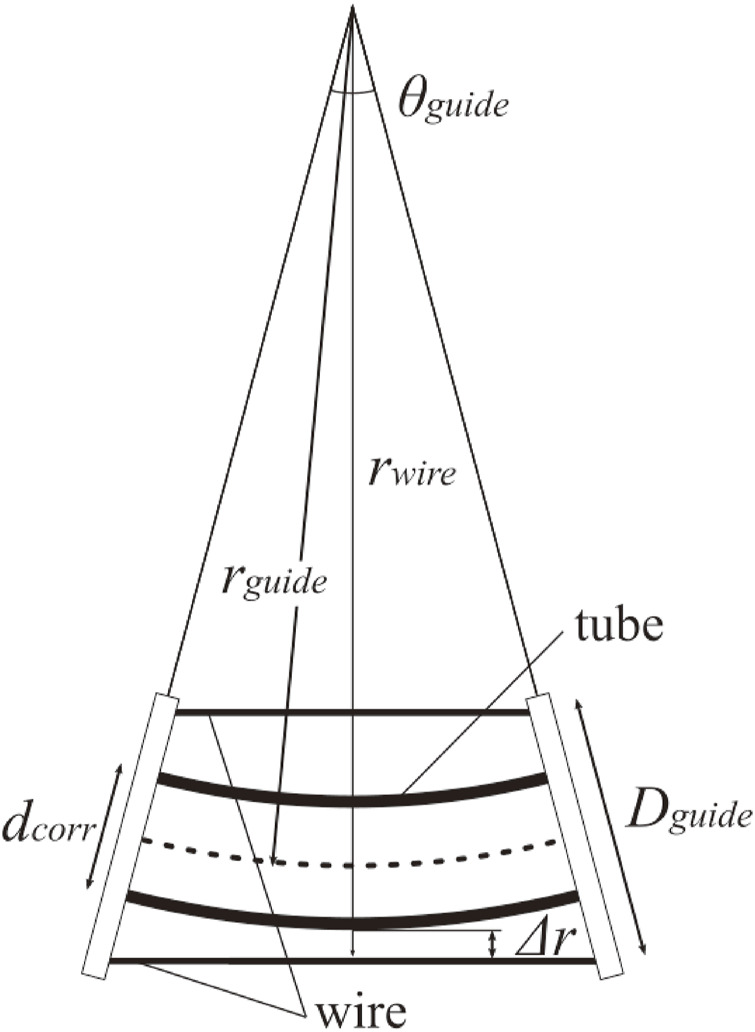
Model of the wire guide.

To implement the mechanism on the DFF, we first designed the diameter of the wire guide for the wire to not touch the body when bending. Assuming that the bend in the body is a circular arc, we consider a geometrical situation of wires and guides for one section (Figure 10). Let *θ*_*guide*_ be the bending angle, *d*_*corr*_ be the outer diameter of the corrugated tube, and *r*_*guide*_ be the bend radius from the bending center to the centerline of the tube. The distance between the wire and tube Δ*r* can be calculated as follows:

$$\Delta r=\left(r_{\mathrm{guide}}+\frac{D_{\mathrm{guide}}}{2}\right)\cos\frac{\theta_{\mathrm{guide}}}{2}-\left(r_{\mathrm{guide}}+\frac{d_{\mathrm{corr}}}{2}\right)$$ (19)

The condition where the wires do not interfere with the tube is Δ*r* > 0. Therefore, once *θ*_*guide*_, *r*_*guide*_, and *d*_*guide*_ are determined, the condition that *D*_*guide*_ should fulfill can be obtained.

We set the wire guide spacing as 200 mm, and the largest body curvature was assumed when the 4 m DFF was bent into a circle. In this case, *d*_*corr*_ = 60 mm, *θ*_*guide*_ = *π*/20 and *r*_*guide*_ = 4000/*π* mm. We get the condition *D*_*guide*_ > 68.6 mm.

**FIGURE 11:**
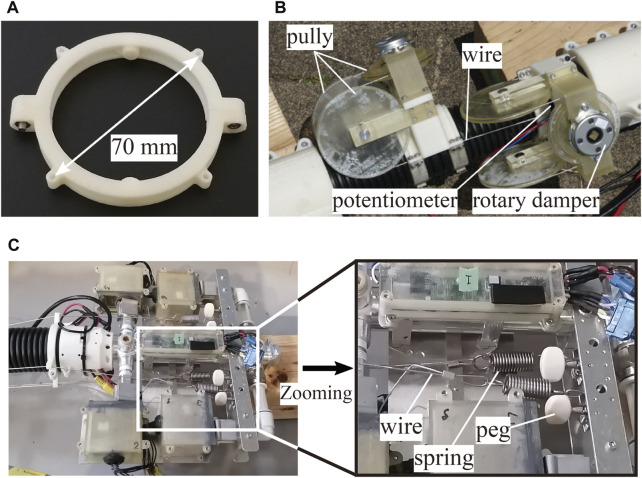
Photos of the developed damping mechanism. **(A)** Wire guide. **(B)** Damping mechanism at the root. **(C)** Tensioner and peg on the head nozzle unit.

Based on the aforementioned discussion, a wire guide is designed, as shown in Figure 11A. The distance between the guides was 200 mm, and the wire spacing was set to 70 mm to minimize the diameter as much as possible.

Figure 11B shows the rotary damping mechanism at the root. The wire threaded from the head was folded back at the pulley to the head again. The pulley was equipped with a rotary damper (FDN-47A-103, *Fuji latex*) with a damper coefficient of approximately 0.31 Nm/(rad/s) (close to the optimum value) and a potentiometer (3590S-4-103L, *Bourns*) that rotated in conjunction with the pulley.

Both ends of the wire were placed inside the head nozzle unit. One end was attached with a spring (wire tensioner spring), and the other end was wound around a guitar peg to adjust the wire tension (Figure 11C). The wire tensioner spring was attached to prevent the wire from loosening during deformations. Because the wire guides were arranged discretely along the body, the whole path length for the wire changed as the robot changed its shape. Particularly, the wire may loosen when the curvature of the body increases. We determined the spring stiffness through a trial-and-error process to prevent the wire from loosening and allow the robot to deform easily. If the spring is too soft, it cannot overcome the friction of the wire guide to eliminate the wire loosening. On the other hand, if the spring is too stiff, the wire is subjected to a large amount of tension when the robot forms a straight shape, preventing the robot from achieving a straight shape. Therefore, the spring constant must be determined to balance these factors.

The guitar peg attached to the wire is assumed to adjust the wire’s tension before each flight. Because the corrugated tube may deform slightly along the longitudinal axis during several flights, it is necessary to adjust the wire tension before each flight. Concretely, we first ensured that the robot’s shape was as straight as possible and then removed any looseness in the wires manually so that all four springs could extend evenly. Finally, we adjusted the spring tension by rotating the pegs to ensure that the extension length of the spring was approximately half of the maximum extension length.

This study has some limitations. While we designed the geometric parameter of the wire guides, we did not perform structural optimization. In future work, the structure of the wire guide should be optimized to increase its durability. In addition, the wire tensioner spring was determined based on a trial-and-error approach. In future work, a precise robot model that accounts for the friction effects of the wire must be developed to determine the feasible spring constant.

### 5.5 Controller with firefighting mode

Because of the left-right symmetry of the nozzle unit, the roll-rotating nozzles can direct outward or inward to generate the same net force. In the conventional controller, the roll-rotating nozzles expel the jets outward because the outward jets increase the range of realizable net force because the jets do not interfere with the nozzle unit itself. However, it was difficult to shoot the jet at the fire source. Thus, in this section, we propose a firefighting mode that expels the jets inward. Although the inward jets decrease the range of the net force due to interference with the nozzle unit, it is easy to shoot the jet at the fire source because the water jets are directed downward of the nozzle unit. This section briefly explains DFF control with the firefighting mode.

The general control method is the same as that of the conventional DFF mentioned in Section 3.2. We modified the calculation method of rotating nozzle angles (nozzle angle optimization) to newly apply the firefighting mode. The brief control diagram is shown in Figure 2C. We set the rotating nozzle angles *ϕ*_*i*1−*i*4_ for nozzle unit *i* as those shown in Figure 1C. We also set the coordinate on the nozzle unit *i* as Figure 1C and represent the net force and torsional torque as ***R***_*i*_ and *M*_*ix*_ on this coordinate, respectively. We calculate *ϕ*_*i*1−*i*4_ using quadratic programming (Ando et al., 2020) to minimize the following function:

$$\begin{array}{ll} \Phi_{i} & =a_{i1}{\left(R_{i1}^{d}-R_{i1}\right)}^{2}+a_{i2}{\left(R_{i2}^{d}-R_{i2}\right)}^{2}+a_{i3}{\left(R_{i3}^{d}-R_{i3}\right)}^{2}+a_{i4}{\left(M_{ix}^{d}-M_{ix}\right)}^{2}\\ & \;+b_{i}\overset{4}{\underset{j=1}{\sum }}{\left(\phi_{ij}-\phi_{ij}^{p}\right)}^{2}+c_{i}{\left(\phi_{i3}-\phi_{i4}-\Delta \phi_{i}\right)}^{2} \end{array}$$ (20)

where $R_{i}^{d}$ and $M_{ix}^{d}$ are the force vector and torsion torque to be realized (determined by the coordinate transformation of $f_{i}^{n}$ and $\tau_{i}^{t}$ ), respectively. $\phi_{ij}^{p}$ is the previous optimized angle *ϕ*_*ij*_. ***a***_*i*_, *b*_*i*_, and *c*_*i*_ are constants and weights of the optimization functions. ***a***_*i*_ determines the preferred accuracy of the realized force and torque. *b*_*i*_ prevents rapid changes in the nozzle direction. *c*_*i*_ tries specifying the angle difference of roll-rotating nozzles to be Δ*ϕ*_*i*_. We note that *ϕ*_*i*1−*i*4_ are determined to fulfil the following moving range as

$$0{}^{\circ}≦\phi_{i1,i2}≦180{}^{\circ},-90{}^{\circ}≦\phi_{i3}≦75{}^{\circ},-75{}^{\circ}≦\phi_{i4}≦90{}^{\circ}.$$

We calculate this quadratic programming for 100 Hz on the microcomputer.

In the firefighting mode, we set Δ*ϕ*_1_ = 15° to ensure that the head nozzle expels the jets inward and downward. Otherwise, we set Δ*ϕ*_*i*_ = −45° to realize the outward injections. In the transition phase, we gradually increased *c*_*i*_ to forcefully change the roll-angle difference and then changed Δ*ϕ*_1_ smoothly from -45° to 15° with time to avoid abrupt changes of expelling directions. After we set Δ*ϕ*_1_ as 15, we gradually decreased *c*_*i*_ to the initial value. The transition to the normal situation is *vice versa*. In the demonstration, we set $a_{i}={\left[0.2 1 1 1\right]}^{T}$ , *b*_*i*_ = 1, initial *c*_*i*_ = *π*/180, and transition *c*_*i*_ = *π*/90 because we wanted to deprioritize the realization of the force in the *x*-direction. We also set the transition time as 5 s with some trails and errors. As other control parameters, we set the non-dimensional D gain of the controller *s* as 10 and 15 for the head and middle nozzle units, respectively.

## 6 Demonstration

At the Fukushima Robot Test Field, firefighting was demonstrated in the Opening Ceremony of WRS2020 Fukushima on 8 October 2021. The detailed procedure of this demonstration is provided in Section 4.2, and the video is available on the internet (World Robot Summit, 2021). During this firefighting demonstration, we could accomplish all the missions to extinguish the fire.

**FIGURE 12:**
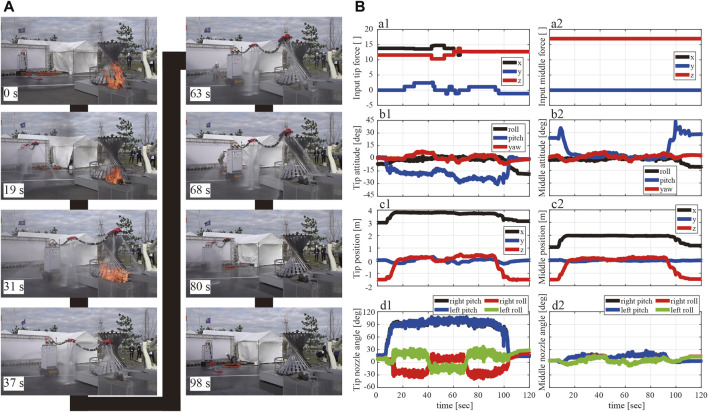
**(A)** Snapshot of the DFF demonstration at rehearsal. **(B)** a1: Head nozzle and a2: middle nozzle show the time response of the force input at $F_{1}^{n}$ and $F_{2}^{n}$ . Black, blue, and red lines correspond to the *x*, *y*, and *z* components of $F_{1}^{n}$ and $F_{2}^{n}$ , respectively. b1: Tip nozzle and b2: middle nozzle show the attitude of nozzle units. Black, blue, and red lines correspond to the attitude (roll, pitch, and yaw angles) with respect to the inertial frame, respectively. c1: Tip nozzle and c2: middle nozzle show the position of the nozzle units. Black, blue, and red line segments represent the *x*, *y*, and *z* components of the nozzle position relative to the root fixed point. d1: Tip nozzle and d2: middle nozzle show the nozzle angle of the nozzle units. Black, blue, red, and green lines correspond to the nozzle angle (*ϕ*_1_, *ϕ*_2_, *ϕ*_3_, and *ϕ*_4_), respectively.

Because we did not capture videos in the demonstration (we concentrated on the demo), this study shows and discusses the practice session results. The video of this rehearsal is available as a supplementary video. Figure 12A shows snapshots of the robot’s behavior during practice. The time count starts when the fire engine starts to supply the water to DFF. The DFF completed the take-off movement 19 s later; subsequently, the active cart moved forward and reached the fire extinguishing point within 31 s. At the fire extinguishing point, the robot finished shifting to the fire extinguishing mode (inward jetting) within 37 s, and the time required to shift to the fire extinguishing mode was 6 s. Fire extinguishing was performed by moving the head position up, down, left and right. Fire extinguishing was finished at 63 s. The time required to extinguish the fire was 26 s. After extinguishing the fire, the DFF directed the roll nozzles of the head nozzle unit outside in 68 s, and the time required to shift to the normal mode was 5 s. Then, the robot moved backward (80 s) and finally landed (98 s).

As shown in Figure 12B, the commanded force (a1 and a2), the postures (b1, b2) and positions to mobile base (c1, c2) of each nozzle unit, and the directions of injection nozzles (d1, d2) are shown as time variation. In Figure 12Ba1, a2, the black line represents the force component in the front-back direction (*x*-direction), the blue line represents that in the left-right direction (*y*-direction), and the red line represents that in the vertical direction (*z*-direction). The commanded constant net force $F_{i}^{n}$ is constant, except during firefighting. During firefighting, the commanded force is changed to adjust the position of the head to direct the injected water to the fire source. In Figure 12Bb1, b2, the black, blue, and red lines indicate the roll, pitch, and yaw angles, respectively. Notably, during the flight phase (19–90 s), the amplitude of posture oscillations is less than 10 deg, while some posture changes occur depending on the commanded force inputs. In addition, even after take-off, the robot did not experience large posture changes because of the appropriate design of the damping mechanism. In Figure 12Bc1, c2, the black lines are the positions in the *x*-direction (front-back) to the root on the mobile base, the blue lines are the y-positions (left-right) to the root on the mobile base, and the red lines are z-positions (up-down) to the root on the mobile base. The head and middle nozzle can successfully float up to the same height as the root on the mobile base (approximately 1.5 m) because the height is zero. The head x-position is almost 4 m, and y-position is almost zero during the flight, which means that the 4 m robot flies in almost a straight line. In Figure 12Bd1, d2, the black, blue, red, and green lines indicate the right-side pitch (*ϕ*_1_), left-side pitch (*ϕ*_2_), right-side roll (*ϕ*_3_), and left-side roll (*ϕ*_4_) nozzles, respectively. The waterproofed servomotors can control the rotating nozzles properly without severe communication failures. Notably, the head nozzle unit can achieve the fire-extinguishing mode by injecting the roll-rotating nozzles inward from 40 to 70 s.

## 7 Lessons learned

The developed DFF system incorporating a mobile base could achieve remote fire extinguishing. This could increase the impact of the robot’s applicability to the actual fire-extinguishing tasks because it contributes to safe operation. In addition, the fire extinguishing mode facilitated precise water shooting to the fire and improved fire extinguishing performance. Conventionally, because the water jets were injected in the outward direction, shooting at the specific point, i.e., the fire source, is difficult for an operator.

There is considerable scope for future developments. Notably, appropriate postures of nozzle units can be used to realize better performance of the DFF. Because the range in which the net force of the nozzle unit can be realized is limited and depends on the robot’s posture, posture selection of the nozzle unit is important to realize the commanded net force. For example, the robot performed better in the demonstration case when the roll postures of the nozzle units were zero during flight. In this demonstration, we determined the feasible postures through a trial-and-error approach. However, in the future, we plan to introduce shape optimization methods to realize the optimal postures for the nozzle units depending on the desired head nozzle positions.

Although the passive damping mechanism effectively stabilizes the levitation, adjustment of the mechanism is time-consuming. We needed to check or adjust the tension of the wires for each flight carefully. If the wire tension is not appropriate, the wire does not move smoothly and may stop due to friction. This changes the flight shape of the robot even under the application of the same commanded forces. This is a concern that must be resolved for practical use. In the future, we plan to propose more advanced controllers to dampen the body oscillations, replacing the passive mechanism. We have already shown the possibility of damping the oscillation without relying on this mechanism. For example, we have found that the flowing water can dampen the higher modal oscillations (Ambe et al., 2022) or that the disturbance rejection controllers can be applied to achieve better damping (Maezawa et al., 2023).

The physical properties of the corrugated tube body changed due to the change in the thermal situation. In the demonstration, the robot could not fly in the same shape as that in the rehearsal, while the commanded inputs were the same. We think that the reason for this phenomenon is as follows: the corrugated tube used as the body became warm because of the direct sunlight during the long standby time before the demonstration, which caused plastic deformation of the body and changed its physical properties. In the future, we will address this problem by choosing a less thermosensitive material. In addition, in terms of control strategy, we plan to estimate and compensate for the plastic deformation by comparing the shapes calculated from the robot model with those obtained from the sensors.

## 8 Conclusion

This study developed and demonstrated a remotely controllable 4 m flying firehose robot, showcased at the WRS2020 opening ceremony. Our approach began with the installation of a mobile base at the robot’s foundation to achieve translational movement by remote control. Subsequently, we re-designed the water channels, adjusting the sizes of nozzle outlets, to obtain enough thrust for aerial flight alongside a fire engine. We also developed nozzle units with a larger movable range (±90°: 1.5 times larger than the conventional nozzle) and waterproofing techniques to improve system reliability. Additionally, we re-designed the passive damping mechanism to ensure better flight stability. Finally, with the developed robot, we successfully extinguished the fire at the firefighting demonstration held at the Opening Ceremony of WRS2020 Fukushima on 8 October 2021, and have learned many lessons through the demonstration. For example, we found that the applied passive mechanism was functional but impractical because it took an extended time period for flight preparation. We also found that the plastic deformation of the corrugated tube caused by heat cannot be ignored in outdoor applications. Based on these lessons learned, we will further improve the robot in future studies. These efforts include improvements in controllers and mechanical designs, such as the damping controller, and finding less thermosensitive materials.

## Data Availability

The raw data supporting the conclusions of this article will be made available by the authors, without undue reservation.
